# Contradictions hindering the provision of mental healthcare and psychosocial services to women experiencing homelessness in Addis Ababa, Ethiopia: service providers’ and programme coordinators’ experiences and perspectives

**DOI:** 10.1186/s12913-023-09810-z

**Published:** 2023-08-01

**Authors:** Kalkidan Yohannes, Yemane Berhane, Hannah Bradby, Sibylle Herzig van Wees, Mats Målqvist

**Affiliations:** 1 SWEDESD, Department of Women’s and Children’s Health, Uppsala University, Uppsala, SE-751 85 Sweden; 2 Department of Women’s and Children’s Health, WoMHeR– Women’s Mental Health during the Reproductive Lifespan, Uppsala University, Uppsala, Sweden; 3 Department of Psychiatry, College of Medicine and Health Sciences, Dilla University, Dilla, Ethiopia; 4 Addis Continental Institute of Public Health, Addis Ababa, Ethiopia; 5 International Child Health and Nutrition- Department of Women’s and Children’s Health, Uppsala University, Uppsala, Sweden; 6 Department of Sociology, Uppsala University, Uppsala, Sweden; 7 Department of Global Public Health, Karolinska Institute, Stockholm, Sweden

**Keywords:** Qualitative research, Mental healthcare, Psychosocial support, Street homelessness, Women of reproductive age, Low- and middle-income countries, Service provider perception, Ethiopia

## Abstract

**Background:**

Mental health conditions are among the health issues associated with homelessness, and providing mental healthcare to people experiencing homelessness is challenging. Despite the pressing issue of homelessness in Addis Ababa, Ethiopia, there is scant research on how service providers address women’s mental health and psychosocial needs. Therefore, we explored service providers’ and programme coordinators’ perceptions and experiences regarding mental healthcare and psychosocial services delivery to women experiencing street homelessness in the city.

**Methods:**

We conducted a descriptive qualitative study with selected healthcare and social support providers and programme coordinators. The study involved 34 participants from governmental and non-governmental organisations in Addis Ababa, Ethiopia. Data were analysed using an inductive thematic approach.

**Results:**

Four themes were derived from the analysis. The first of these was “divergent intentions and actions”. While service providers and programme coordinators showed empathy and compassion, they also objectified and blamed people for their own homelessness. They also expressed opposing views on mental health stigma and compassion for these people. The second theme addressed “problem-solution incompatibility”, which focused on the daily challenges of women experiencing homelessness and the types of services participants prioritised. Service providers and programme coordinators proposed non-comprehensive support despite the situation’s complexity. The participants did not emphasise the significance of gender-sensitive and trauma-informed care for women experiencing street homelessness in the third theme, “the lack of gendered and trauma-informed care despite an acknowledgement that women face unique challenges”. The fourth theme, “mismatched resources,” indicated structural and systemic barriers to providing services to homeless women.

**Conclusions:**

Conflicting attitudes and practices exist at the individual, organisational, and systemic levels, making it challenging to provide mental healthcare and psychosocial services to women experiencing homelessness. An integrated, gender-sensitive, and trauma-informed approach is necessary to assist women experiencing homelessness.

**Supplementary Information:**

The online version contains supplementary material available at 10.1186/s12913-023-09810-z.

## Background

There has been an alarming increase in homelessness over the past ten years, and its consequences affect every country [1, 2]. According to the United Nations Human Rights Council (UNHRC), “homelessness is a profound assault on dignity, social inclusion, and the right to life” [3]. The United Nations Human Settlements Programme (UN-Habitat) estimates that 1.6 billion people live in inadequate housing, and approximately 15 million are homeless yearly [4]. Furthermore, developing countries have a large number of individuals experiencing homelessness [1, 2]. In 2018, it was reported that more than a billion people lived in shantytowns or informal settlements, with 80% of these individuals residing in developing countries [1].

Homelessness can be defined differently in different countries and contexts [5], and Ethiopia does not have a consistent definition of homelessness [6]. This makes it challenging to address the issue effectively and provide support to those who need it most [5]. This study focuses on people who live on the streets alone or with their families and are homeless.

Homelessness exacerbates mental health problems among women experiencing homelessness and increases the risk of mental illness and substance use disorder (SUD) [7]. The experience of homelessness can have a devastating effect on a woman’s psychological and physical health [7]. Studies have shown a link between mental illness and homelessness [7–9]. A recent umbrella review shows high prevalence rates of mental health conditions among people experiencing homelessness [10].

According to the findings of a cross-sectional study among women experiencing homelessness, parenting status is strongly associated with depression or post-traumatic stress disorder (PTSD) [11]. According to the authors’ report, mothers experiencing homelessness are highly susceptible to substance abuse [11]. The prevalence rates of depression and PTSD are higher among women who have been homeless for two or more years [11]. According to a recent review, 29.1 per cent to 41.4 per cent of homeless women experience PTSD [7].

According to a qualitative study conducted in Addis Ababa, Ethiopia, various factors related to living on the streets contribute to the onset and continuation of problematic substance use among women experiencing homelessness [12]. As per the research findings, women who live in shelters and struggle with substance abuse are at risk of premature death, prolonged addiction, and unfavourable health consequences [12]. Additionally, this population has a high prevalence of substance abuse [13].

Various factors, including violence, contribute to mental health and psychosocial problems among women experiencing homelessness [14]. The relationship between violence against women and mental health conditions is well-documented [15]. According to a multicentre randomised controlled trial, women experiencing homelessness are more likely than men to report being assaulted physically or sexually in the past six months [14]. Women experiencing homelessness are frequently victims of violence [15].

Women who have experienced violence are at higher risk of mental health conditions and sexually transmitted diseases [16], as well as other non-communicable trauma-related conditions such as chronic pain [17]. According to the study by Wilson and his colleagues [18], these women have difficulty accessing appropriate medical care. Furthermore, women experiencing homelessness face challenges such as prostitution [13], deteriorating health during pregnancy [19], and stressful life events [20].

Mental health and psychosocial support (MHPSS) is a model that encompasses any assistance that promotes or protects psychosocial well-being and prevents mental illness [21]. MHPSS is the provision of services that address psychiatric disorders such as depression, anxiety, and PTSD [21, 22]. The scope of MHPSS extends far beyond the treatment of mental health conditions to support the psychosocial well-being of individuals and assist in establishing relationships with their family and community members, helping them cope with personal and practical challenges [22].

Nevertheless, studies have also shown that providers face challenges when providing services to people experiencing homelessness [23–26]. Moreover, health insurance issues, issues with discharge planning, structural barriers, a lack of coordination between services, prejudice towards people experiencing homelessness, and inadequate mental health outreach can also hamper mental health service provision to people experiencing homelessness [23, 26].

Ethiopian national health policy has long placed a high priority on mental health [27, 28]. A mental health programme has been established within the Non-communicable Disease (NCD) case team of the Disease Prevention and Control Directorate [28]. As of 2019, the programme operates separately from the NCD case team. In addition, the Ministry of Health has developed a National Mental Health Strategy for 2020–2025 [28].

According to the Ministry of Health’s national health workforce update for 2019, mental health professionals comprise 0.26 per cent of the national health workforce [28]. In terms of mental health workforce per 100,000 of the population, Ethiopia’s mental health workforce is at the same level as that of low-and middle-income countries (LMICs), at less than 2 per cent, as reported in the WHO Mental Health Atlas 2017 [29], which is far below the level recommended by WHO [28]. Furthermore, there are only 0.1088 psychiatrists per 100,000 people in the country, compared with 0.898 psychiatrists recommended by the WHO [30, 31].

Furthermore, Ethiopia lacks mental health legislation, as do most LMICs [32, 33]. As part of mental health legislation, mental health services should be integrated into primary healthcare services for all individuals with mental illness [34]. Further, the legislation is necessary to improve access to healthcare for people with mental illnesses and to provide high-quality mental healthcare [33].

Across most parts of the world, there is a gap between the availability of mental health services and the demand for them, commonly referred to as the “treatment gap” [34]. In Ethiopia, to bridge this gap, the Mental Health Gap Action Programme (mhGAP) [35] and PRIME (Programme for Improving Mental Health Care) [36] are integrated with mental health services in primary care programmes. Although this is critical to address the service gap, mental health structures fail to address the mental healthcare needs of individuals experiencing homelessness [37].

Ethiopia’s Ministry of Labor and Social Affairs is the primary provider of general services and spasmodically works with healthcare organisations [38]. However, there are other agencies that provide services [38, 39]. For example, other governmental organisations, charity institutions and a few public hospitals have signed memoranda of understanding, allowing them to collaborate with the Labor and Social Affairs Bureau to provide services to institutionalised individuals experiencing homelessness [40].

Ethiopia has experienced conflicts, internal displacements, and an increased number of individuals experiencing homelessness [41, 42], resulting in a significant shortage of social services and mental healthcare for individuals experiencing homelessness [42].

Although homelessness negatively influences mental health and psychosocial well-being, little is known about the state of mental health and psychosocial services in Ethiopia from the perspective of service providers. To our knowledge, this topic has not been researched in Ethiopia. Thus, this study aimed to examine how service providers and programme coordinators perceive and experience the delivery of mental healthcare and psychosocial services to women experiencing homelessness.

## Methods

### Study setting and period

We collected data in Addis Ababa, the capital of Ethiopia, between August and September 2021. Ethiopia is located in the north-eastern part of Africa, also known as the Horn of Africa [43]. Sudan and South Sudan border it to the west, Eritrea and Djibouti to the northeast, Somalia to the east and southeast, and Kenya to the south. Ethiopia has the second-largest population in Africa, following Nigeria [43]. The sex ratio is nearly equal between men and women, and women of reproductive age makeup 23% of the population [44].

The mhGAP is integrated into health centres across the country [35]. However, the Ministry of Health, Ethiopia report 2019 revealed that merely 26% of healthcare facilities offer mental health services as part of their overall healthcare provision [44]. In Addis Ababa, individuals can access mental health services through various facilities. St Amanuel Specialized Mental Hospital is a dedicated psychiatric hospital with 268 beds. Eka Kotebe General Hospital, the second-largest public hospital in the city, offers inpatient mental health services and has 150 beds. Additionally, 25 hospitals within the city provide outpatient mental health services [28].

### Study design and study participants

This study employed a descriptive qualitative research design. We conducted semi-structured interviews with 34 care providers, service coordinators, and organisation managers in Addis Ababa, Ethiopia. Twenty-four participants were employed in public health institutions as healthcare providers, programme leaders, coordinators, focal persons, and psychologists. The other eight stakeholders were programme coordinators and leaders of non-governmental organisations. Furthermore, two members of the Mental Health Service Users’ Association (MHSUA) participated. We followed the Standards for Reporting Qualitative Research and the Consolidated Criteria for Reporting Qualitative Research [45, 46].

### Sampling and interview procedure

We interviewed individuals with experience in providing mental healthcare and psychosocial services, coordinating and managing those services, and managing mental health service users’ associations and those with regular contact with individuals experiencing homelessness. The purposive selection of participants was based on their experience in providing services and organising programmes, along with their professional backgrounds. In addition to KY, six research assistants with master’s degrees in mental health and public health conducted interviews in Amharic, utilising a semi-structured interview guide, without permitting anyone else to listen in on the interviews. The topic of how they meet the mental health needs of people experiencing homelessness was discussed, such as mental health and psychosocial services (current situation, challenges, needs, policies, guidelines, manuals), obstacles to service provision, and support for women experiencing homelessness. Each session lasted between 35 and 60 minutes (mean = 48 min). We used the audio recordings to collect the data. The authors terminated data collection once they perceived data saturation had been reached.

### Data analysis

Research assistants and KY conducted, transcribed, and translated the Amharic audio data into English. Braun and Clarke’s six phases of thematic analysis were applied using an inductive approach [47]. Within the emergent design process, we selected concepts based on the analysis of our interviews. In the first phase, KY read each interview several times and discussed the preliminary impressions with MM. In the second phase, KY read and generated initial codes and discussed coding trees with MM. We managed and analysed the data using NVivo V.12 software. The codes were categorised into candidate themes in the third phase. MM and KY examined the candidate themes based on the codes and data. In the fourth phase, we reviewed the themes. In the fifth phase, KY and MM defined the themes, provided written comments, and met to discuss them. In response to these discussions, MM and KY redrafted the themes. In the final stage, both revised and discussed the final draft until a final version was reached. A 15-point checklist of criteria for an excellent thematic analysis process was used [47].

### Reflexivity statement

Personal, interpersonal, methodological, power dynamics, and contextual factors [48, 49] are essential to understanding the research process. Regarding the nature of the studied population–researcher relationship, researchers with mental health and public health backgrounds interviewed the participants, including the first author KY (MSc in Integrated Clinical and Community Mental Health- ICCMH). All interviewers were from the local context, and this, in combination with their previous professional experience, could introduce pre-conceived ideas about the topic of investigation. Thus, they were able to take an outsider’s perspective while at the same time having adequate experience to understand the context. However, a team of two men and three women of varying seniority co-authored this paper. The interdisciplinary research team consisted of a Professor of Global Health (MM) and Public Health and Epidemiology (YB), a professor of Sociology (HB), and a post-doctoral researcher in anthropology (SHvW). All of them are currently employed as researchers and academics. In addition to extensive experience in qualitative research, four of the research team members have conducted fieldwork in Africa, Europe, and Asia. However, they had no contact with the research participants.

This study was conducted as part of KY’s study on Global Health. Participants were aware of this study context. Before the study, the first author (KY) worked as a clinician and academic in psychiatry. Her experience as both a clinician and an academic in the psychiatry department has directed her towards several research avenues related to mental health. In addition, the primary author, as well as three research assistants, are mental health professionals. They worked at one of the hospitals selected for data collection during our master’s training, so some participants were once our colleagues and supervisors. Hence, we saw ourselves as team members and took an insider perspective. However, there were challenges associated with this, influencing the research. One thing we struggled with during the interviews was disclosing our status as mental health professionals and my (KY) status as a PhD student. In a few interviews, some participants remarked, ‘You know how it is when you have encountered people and family experiencing stigma, right?’; ‘You know how the resources are allocated for mental health services, right?’; ‘You know how the government neglects mental health?’. We wonder whether they thought we (the data collectors) were judging them and if they saw us as censorious outsiders.

It was possible that the interviewers’ familiarity with the context could have affected their interactions with the participants as colleagues and former students. Furthermore, while we selected a methodological approach best suited to addressing the research question, it was not the sole option. While doing so, we meticulously assessed and accounted for the influence of our professional knowledge and prior experience throughout the study’s data collection, analysis, and write-up stages.

## Results

### Characteristics of participants

This study involved care providers from diverse professional backgrounds, including clinical psychologists, counselling psychologists, health officers, nurses, social workers, sociologists, public health experts, social science professionals, mental health professionals (psychiatrists, psychiatry nurses, MSc in ICCMH), and medical doctors. We interviewed 34 participants, including men and women aged 25–61, as well as seniors and recent graduates working as care providers and program coordinators in mental health and psychosocial services. The program coordinators represented various backgrounds, including management, medicine, leadership, public health, psychology, and mental health. Apart from the team leaders, there were also founders of charity organisations and individuals responsible for leading MHSUA (Table 1).

**Table 1:** shows the socio-demographic characteristics of participants in Addis Ababa, Ethiopia, 2021 (n = 34)

Variables	Categories	Number of participants
**Age of the participant**	< 30	6
	30–39	17
	≥ 40	11
**Gender**	Male	21
	Female	13
**Profession**	Psychiatrist and a general practitioner (GP)	4
	Management and leadership	9
	Psychiatry and comprehensive nursing	5
	General psychology (BA)	6
	MSc in mental health (ICCMH)	2
	Social Work (MSc) and social science (BA)	5
	Clinical, counseling, and social psychology (MSc)	3
**Organization**	Ministry of Women and Social Affairs	1
	Addis Ababa City Administration Labor and Social Affairs Bureau	4
	Addis Ababa City Administration Health Bureau	3
	Eka Kotebe General Hospital	4
	Mekedonia Charity Association	4
	Gergesenon Charity Organization	4
	Born Again Rehabilitation Center	1
	Bureau of Women Children and Youth Affairs	1
	Addis Ababa Mayor Office	1
	St. Amanuel Mental Specialised Hospital and other charity organisations	11
**Position**	Organisation founder/initiator	3
	Administrator	2
	Association president	1
	Team leader/focal person	4
	Director	4
	Programme coordinator	4
	Clinician/counselor	5
	Staff	8
	Assistant head	1
	Executive committee of the association	1
	Volunteer/service facilitator/clinical service	1

**Fig. 1:**
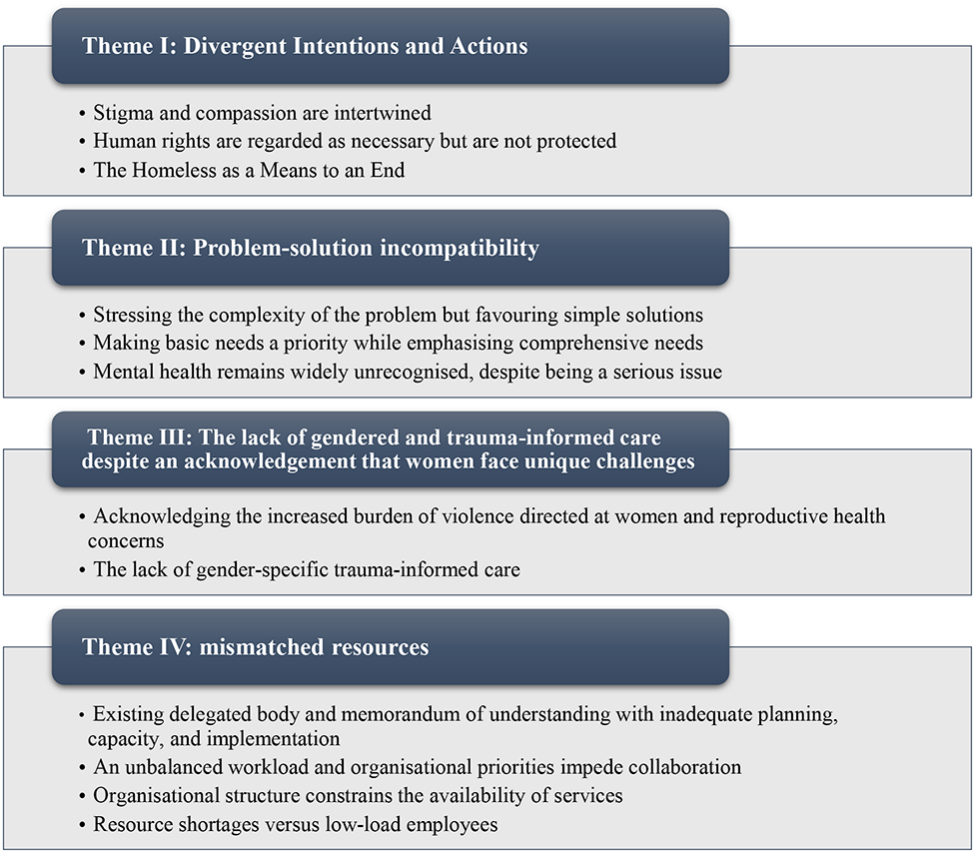
Themes and subthemes from qualitative analysis

During the interview, providers and program coordinators discussed their perspectives and experiences regarding delivering mental health and psychosocial services to women experiencing homelessness. A particular focus was placed on the situation, barriers, and facilitators of mental healthcare and psychosocial support for individuals experiencing homelessness in Addis Ababa, Ethiopia. The following four themes emerged from twelve subthemes (Fig. 1).

1. “Divergent intentions and actions”; (2) “Problem-solution incompatibility”; (3) “The lack of gender-neutral, non-trauma informed care while acknowledging women’s unique challenges”; and (4) “Mismanaged resources”.

### Theme 1: divergent intentions and actions

The service providers and program coordinators highlighted ongoing interventions, homelessness conditions, mental healthcare, and psychosocial support for women experiencing homelessness. Several participants expressed concern about the daily violations of human rights experienced by these individuals. Despite this, people experiencing homelessness are sometimes viewed as a potential security threat.

Several participants described how politicians plan and implement campaigns without regard for continuity of care. City administrators provided short-term rehabilitation programs; however, as participants noted, once these organisers achieved their organisational goals, they did not continue to assist people experiencing homelessness. In addition to failing to consider the predicaments of individuals experiencing homelessness after reaching their organisational goals, these organisers displayed poor sustainability practices. Participants pointed out that they provided short-term assistance to people experiencing homelessness despite knowing they were facing a complex and extensive problem. The sub-themes are described below.

#### Stigma and compassion are intertwined

The study participants reported that the presence of a gap in awareness in the community regarding mental health contributed to stigmatisation and discrimination against persons with mental illness. Participants discussed the stigmatisation experienced by those living with mental illnesses and homelessness within the community and the family. In contrast, the participants discussed the presence of sympathy in the community for panhandlers, persons with disabilities, and women with dependent children. The following excerpt illustrates the stigma associated with mental health issues.

*People with mental health conditions are stigmatised and discriminated against in the community. The common misconception is that mental illness results from sin, a curse, or evil (IDI#28, healthcare provider, charity).*

Several mental health professionals have stressed that homelessness and mental illness result in a double discrimination burden. According to the participants, individuals with mental health conditions are often subject to discriminatory behaviour within their communities and families. The experience of this stigmatisation inhibits them from seeking medical care. It might contribute to non-adherence to treatment and chronic mental illness. This issue may also hinder the delivery of services.

Some participants expressed concern that individuals experiencing homelessness may threaten their communities. Some participants noted, for example, that these individuals might steal from the community. Participants stressed that neither the community nor the legal system could empathise with people experiencing homelessness. The service providers also addressed issues related to stereotyping that classified every person living on the streets as a criminal and a drug user. The extract below illustrates how the community and government stereotype individuals experiencing homelessness as criminals.

*In particular, women experiencing homelessness face two distinct challenges and insecurities.*

*Besides being exposed to cruelty and violence, many homeless individuals are viewed as criminals by the community and law enforcement bodies (IDI #29).*

The participants discussed that service delivery barriers might come from people experiencing homelessness. According to a rehabilitation officer, some homeless individuals do not wish to complete rehabilitation and treatment.

*Our examination of the fundamental interests of individuals experiencing homelessness reveals that they aspire to earn a living in any way possible. They prefer to make more money than to live in a shelter, regardless of whether they live on the streets or elsewhere. Furthermore, I do not believe rehabilitation becomes successful or effective unless people experiencing homelessness are willing to accept it. Sometimes, homeless individuals do not complete rehabilitation programs and end up on the streets (IDI #2, social rehabilitation provider, government institution).*

#### Human rights are regarded as necessary but are not protected

The participants pointed out that although the number of people experiencing homelessness and their burdens have increased recently, homelessness and mental health issues have been neglected for quite some time. Furthermore, a gap in community awareness of mental illness has resulted in discriminatory practices and inhumane treatment of individuals with mental illnesses and those experiencing homelessness. One challenge of service delivery the participants mentioned was the absence of mental health legislation that promoted health equity and healthcare access while protecting the rights of every individual with a mental illness.

*Other countries have a mental health law (the mental health act) that outlines the rights of people with mental illnesses, while Ethiopia does not have one. Sometimes, we hesitate to assist homeless individuals with mental health conditions who have been seriously injured or damaged on the streets (IDI #7, service provider, government agency).*

Participants also noted that there are few champions of mental health. They discussed the need to promote awareness concerning mental health conditions and improve society’s perception of mental illness. The participants discussed the presence of a few advocates and bloggers that use social media to spread awareness of mental health issues.

*Currently, a psychiatrist promotes mental health awareness on his social media platform and is one of a few national advocates for mental health (IDI #29, service provider, government agency).*

A few years ago, one campaign focused on mental healthcare and psychosocial support was conducted on the streets of Addis Ababa. Several participants reported that St. Amanuel Mental Specialized Hospital and the government were responsible for developing and implementing it. However, they identified structural deficiencies that prevent these services from being provided.

On the other hand, participants also pointed out that the legal system does not protect those with mental illnesses and those experiencing homelessness. Their rights and privileges were often different from those living in other circumstances. As a result, they could not receive the support and assistance they required. One participant brought up the limited availability of legal aid and the shifting system for people living on the streets:

*Several cases of abuse have been reported against them (women experiencing homelessness). However, the police do not want to listen to their complaints. Most of the time, they (legal bodies) do not wish to assist them (people experiencing homelessness). Moreover, they (legal bodies) are more inclined to blame people experiencing homelessness rather than listen to them (#IDI 22, service provider, charity).*

#### The homeless as a means to an end

Most participants expressed concern regarding unsustainable support provided to people experiencing homelessness, including one-time-only campaigns, periodic funding, and a lack of attention to continuity. In addition, participants described that women experiencing homelessness received limited political support. In the participants’ opinion, a single campaign was insufficient to resolve the problem. Participants strongly emphasised the importance of structured services integrated into the healthcare system through financial support.

*Establishing an appropriate financial system would answer the questions of who should fund them and how they should be provided (IDI#8 healthcare provider at the governmental organisation).*

During the interview, a participant expressed concerns about Ethiopia’s current pandemic and political upheaval. The participant also pointed out potential obstacles that could hinder sustainable programs:

*COVID-19 cases and Ethiopia’s current political instability make the campaign unlikely to continue (IDI #12, program coordinator, charity organisation).*

### Theme 2: problem–solution incompatibility

Service providers and program coordinators discussed the challenges faced by women experiencing homelessness. The discussion focused on unmet healthcare needs, available interventions, and mental health concerns. From the interview, three sub-themes emerged, drawing attention to specific areas of concern.

#### Stressing the complexity of the problem but favouring simple solutions

Study participants agreed that women who live on the streets experience violence, health problems, and problematic substance use. They discussed the risks associated with childbearing, unsafe abortion practices, and risky sexual behaviour. The participants described additional concerns about road traffic accidents (RTAs), sudden death on the street, and problematic substance use.

*Women experiencing street homelessness face financial hardship, abuse, and isolation. Women’s insecurities include unwanted pregnancies, diseases, and drug abuse (IDI #2, social rehabilitation provider, government organisation).*

Most participants agreed that women experiencing homelessness are rising. They provided several contributing factors, for example, housing issues, internal displacements, women trafficking, domestic violence, and vulnerability to homelessness due to economic and social inequalities. The following extract highlights the problem:

*There has been an increase in the number of women experiencing homelessness. As a result, the number of internal migrants has also increased, particularly from rural southern areas to Addis Ababa (IDI#15, program coordinator, government organisation).*

It was noted that participants placed a lot of emphasis on homelessness’ impact on mental health. They discussed the close connection between mental health conditions and homelessness.

*There is also a link between homelessness and psychological problems. Several health-related issues exist, probably more than I can list. Mental health disorders are a leading cause of homelessness (IDI #18, mental healthcare provider, government organisation).*

#### Making basic needs a priority while emphasising comprehensive needs

When discussing the complex mental, physical, and social challenges women experiencing homelessness face, participants agreed that approaching mental health and psychosocial issues from a multidisciplinary perspective is imperative.

*Mental health treatment is multidisciplinary; we provide both medical and psychosocial support. Managing mental health issues requires the collaboration of psychologists and psychiatrists (IDI #31, service provider, governmental organisation).*

Participants in the study noted that mental health disorders are typically treated with medication, psychotherapy, and social support for the general population. Service providers, such as psychiatrists, clinical psychologists, social workers, rehabilitation officers, medical doctors, and counsellors, discussed various treatment approaches for those with mental health conditions, substance use disorders, or trauma-related conditions. Participants also stressed the importance of family therapy in addition to individual counselling. Family members are known to play a crucial role in assisting patients to cope with stressful situations. Nevertheless, when it came to women experiencing homelessness, the participants indicated that fulfilling basic needs were more critical than such comprehensive management. One participant explained why basic needs must be prioritised over comprehensive mental health services for institutionalised homeless individuals:

*I agree that several problems need to be addressed. However, there is a high demand for basic needs, such as food, water, clothing, sleep, and shelter. Yes, the basics are necessary. However, they (people experiencing homelessness) also face economic difficulties (IDI #7, service provider, government organisation).*

Participants expressed that addressing these individuals’ mental health needs could be challenging without ensuring their basic needs are met. Their primary concern was that without access to shelter, food, water, and other necessities, it becomes difficult for clients to adhere to medication regimens or prioritise treatment for their conditions.

*Providing mental health services is nearly impossible without adequate shelter, food, and security. Food and shelter are essential for medication adherence. In addition, the shelter simplifies access to medicine (IDI #33, Program Coordinator, charity organisation)*

#### Mental health remains widely unrecognised, despite being a serious issue

Participants emphasised that mental health issues and mental health services were neglected issues. Furthermore, they highlighted the importance of this alarming global issue for public health. The importance of integrating mental health services into primary care centres was recognised. Participants expressed concern about the lack of tailored mental health services available to individuals experiencing homelessness. According to participants, non-governmental organisations primarily focus on preventing and treating non-communicable and selected communicable diseases. At the same time, they indicated that mental health services remain underfunded compared to other health programs.

*Insufficient budgets are allocated to mental health services, and these services are not given enough attention. Our office does not place a high priority on mental health in our bureau. The government sector has difficulty obtaining sufficient funding for mental health programs (IDI #15-program coordinator, government organisation).*

The participants indicated that charitable organisations provide mental health services to individuals experiencing homelessness; they emphasised the importance of these organisations’ efforts. A few healthcare providers and coordinators disagreed with the lack of focus on mental health services for people experiencing homelessness. An extract from a mental health professional is provided below:

*The Mekedonia charity organisation provides various services, including mental health and psychosocial support. The organisation assists homeless individuals with mental health conditions (IDI#7, service provider, government organisation).*

### Theme 3: The lack of gendered and trauma-informed care despite an acknowledgement that women face unique challenges

Participants also discussed the mental health consequences of women’s reproductive health issues and violent victimisation. However, they did not acknowledge the lack of gender-specific trauma-informed care for women experiencing homelessness as a pressing issue. The sub-themes are described in detail below.

#### Acknowledging the increased burden of violence directed at women and reproductive health concerns

Psychiatrists, clinical and community mental health professionals, and clinical and counselling psychologists highlighted the mental health and reproductive effects of traumatic and abusive experiences. The service providers paid particular attention to women-distinct challenges, such as unwanted pregnancies, unsafe abortions, violence, and insecurity associated with raising children.

*There are several psychological and physical consequences associated with sexual abuse. In particular, rape has a significant impact on women. Furthermore, sexual abuse may increase their risk of unintended pregnancy, HIV-AIDS, and other sexually transmitted diseases (IDI #12, Healthcare provider, government organisation).*

According to one participant, women living on the streets often experience marginalisation because of their gender. This misapprehension of those in their communities towards women experiencing homelessness is described as follows:

*Women experiencing homelessness encounter marginalisation due to their gender and the misperceptions of those in their communities towards women experiencing homelessness (IDI #1, social rehabilitation officer, government agency).*

#### The lack of gender-specific trauma-informed care

Participants from the mayor’s office, the city’s Labour and Social Affairs office, and the Ministry of Women and Social Affairs discussed the services provided by different sectors to people experiencing homelessness. To begin with, rather than returning them to their original locations, they place them in rehabilitation centres to treat their addictions. Only institutionalised women with lived experiences of homelessness are provided with food, shelter, medical treatment, and psychological and social support as part of this program. A variety of life skills and techniques were taught by the Labour and Social Affairs office. In addition to assisting women with lived experiences of homelessness to build a strong mentality, this program aimed to prepare them for the future. It has been reported that many individuals have returned to their villages, towns, or regional cities of origin in recent years.

Moreover, the office categorises institutionalised women with lived experiences of homelessness according to their educational level and age. In this way, they were assisted in securing employment and providing for their families. As a result, they could work without obstacles and become productive citizens.

*Microfinance programs enable institutionalised women with lived experiences of homelessness to obtain a photocopy machine. We collaborate with microfinance institutions through our office (the Labour and Social Affairs Bureau) to train institutionalised women. Additionally, we connect women with lived experiences of homelessness with local employment opportunities and rehabilitation centres so that they may earn a living (IDI #24, program coordinator, government organisation).*

Even though participants discussed reproductive health, violence, and the impact of street life on women, they did not discuss the importance of trauma-informed and gender-responsive care.

### Theme 4: mismanaged resources

Service providers and coordinators state that several structural and systemic barriers hinder providing mental healthcare and psychosocial services to women experiencing homelessness in Addis Ababa. The following subthemes are related to the central theme.

#### Existing delegated body and memorandum of understanding with inadequate planning, capacity, and implementation

According to the experiences of the four participants from the Labour and Social Affairs office of Addis Ababa city administration, despite their responsibility for overseeing the issues of women experiencing homelessness, this governmentally mandated office still struggles to provide mental health and psychosocial services to homeless individuals.

*Several individuals experiencing homelessness have requested assistance over time; however, the office is becoming limited in providing that assistance (IDI #4, social rehabilitation provider, government organisation).*

Participants highlighted the shortcomings of intervention planning practices and the gaps in the intervention process. Furthermore, they noted that a lack of engagement among actors resulted from poor campaign design, implementation gaps, and planning failures. Participants pointed out that the problem persists despite the office’s involvement.

*There has been an increase in the number of homeless people in Addis Ababa due to migration and displacement from nearby towns. Although support has been provided, the problem persists over time (IDI #3, social rehabilitation officer, government organisation).*

Most care providers and program coordinators emphasised that psychologists, social workers, and healthcare workers employed by government agencies provide mental health services to individuals experiencing homelessness in charity institutions. Participants noted that memoranda of understanding had been signed between charitable organisations and governmental institutions.

*St. Amanuel Mental Specialized Hospital provides psychotropic medications and mental health professionals. Additionally, St. Peter Hospital offers medical care for homeless individuals with the support of our charitable organisation. We often receive assistance from non-governmental organisations (IDI #20, social workers, and charitable organisations).*

However, participants underscored the lack of planning, inconsistent coordination, and insufficient capacity to address the increasing pattern of homelessness and mental illness.

#### An unbalanced workload and organisational priorities impede collaboration

According to Labour and Social Affairs Bureau participants, a lack of balance in the workload has led to a substantial amount of responsibility and burden borne by the office dealing with homelessness. According to the participants, the Labour and Social Affairs Bureau handles most social support activities. In the interview, participants expressed concern that the Office of Labour and Social Affairs failed to emphasise sharing responsibilities and assessment of ongoing activities. The insufficient level of collaboration between actors prevents individuals experiencing homelessness from receiving comprehensive psychological support, as demonstrated by these quotes:

*Neither the government nor non-governmental organisations have conducted joint assessments. A slower pace of rehabilitation has been observed than expected (IDI #2, social rehabilitation officer, government organisation).*

Participants emphasised that mental health and psychosocial services provided to homeless individuals were inadequately structured and underfunded. Following the COVID-19 pandemic, service delivery became complicated due to imbalanced responsibilities between actors, differences in institutional priorities and scope, and resource shortages.

*There is a shortage of resources in rehabilitation centres. Several other actors entrust their responsibilities to the Bureau of Labour and Social Affairs (IDI #4, program coordinator of the program for social rehabilitation, government organisation).*

According to several mental health professionals from public hospitals, the homeless-focused mental health campaign was not sustained due to a lack of financial support and cooperation from other organisations.

Psychiatrists, psychologists, social workers, nurses, and health officers were all involved in the campaign. Participants who worked with the psychiatrist who led the campaign expressed appreciation for his leadership. They also emphasised that the campaign positively influenced homeless individuals with mental health issues. A participant emphasised this point.

*Following the general assessment of street homeless individuals’ conditions, we (staff at Amanuel Mental Health Specialized Hospital) provided cleaning, health assessments, and psychiatric assistance. It was a successful campaign. Despite this, the hospital was under considerable pressure. The hospital staff demonstrated a high commitment and dedication to the campaign. Nevertheless, the campaign was halted by changes in management at other institutions. Although the project could not proceed, there was a positive outcome (IDI #33, service provider, government organisation).*

The participants discussed their organisation’s priorities and the scope of their services. It was emphasised that the priorities of government and non-government organisations differed. A participant stated that his organisation targets the following groups:

*Our mission is to assist seniors and people with disabilities. A primary focus of the Mekedonia Humanitarian Association is to provide shelter, clothing, food, and other necessities to elderly and disabled people who have no other means of supporting themselves (IDI #28, program coordinator, charity organisation).*

#### Organisational structure constrains the availability of services

According to the participants, the Ministry of Health has integrated the WHO’s Mental Health Gap Action Programme (mhGAP) to expand services for mental illnesses, neurological disorders, and substance abuse disorders into primary care. Nonetheless, most participants expressed concern about a lack of a healthcare system that addresses the mental health needs of individuals experiencing homelessness. They also expressed concern about the gap in health insurance inclusivity. According to a participant, the following service gap exists:

*The mental health issue seems to be less of a concern among stakeholders. In addition, homeless individuals have limited access to mental health services. Furthermore, it can be challenging for mental health professionals to provide mental health services without an inclusive healthcare system (IDI #34, service provider, government organisation).*

Participants noted that the country’s economic and political conditions might make it difficult for organisations to provide mental health services to people experiencing homelessness.

*The current economic situation is characterised by high inflation, and political issues have further complicated matters. Those living on the streets face a particularly challenging problem. As a result of the country’s current state, other people who may be able to assist them (street homeless women) are affected. Their situation has worsened over the past few years (IDI #9, service provider, government organisation).*

According to the charity organisation’s program coordinator, several challenges involve moving homeless individuals from the streets to institutions. The participants also accentuated that there is no community-based or legal framework to address the issue, and no precise mechanism exists to expedite their moving from the streets.

*As we attempted to relocate homeless women from the streets, the community frequently asked, ‘what are you intending to do with her?’ Other questions included, ‘For what purpose are you relocating her?’ This sparked controversy, and we were arrested as a result. It was challenging to convince the community that we were taking people experiencing homelessness to a better place (a charity organisation). For this reason, we requested permission from the police station each time we performed this task (IDI #22, program coordinator, charity organisation).*

As stated by another participant from the government organisation, the organisation’s scope and priority are critical elements of task sharing.

*I believe mission and vision are essential components of scope. We provide mental health treatment to patients who come to our facility with mental health problems. Despite this, we collaborate with other organisations. Our hospital organised a campaign to assist homeless mental health patients. Furthermore, we provide medication support to charitable organisations. Nevertheless, the number of mental health professionals sent to charity has decreased over the past few years. The hospital’s involvement in mental health services for homeless individuals is limited. Our limited resources make it impossible for us to provide comprehensive healthcare outside the hospital (#8 healthcare provider, government organisation).*

#### Resource shortages versus low-load employees

Participants discussed the challenges associated with delivering mental health and psychosocial services to people experiencing homelessness created by limitations in organisational resources, namely, a shortage of mental health professionals and limited budget allocations for mental health services. Participants in the survey reported a low demand for mental health services in Addis Ababa’s health centres, which reduces the workload of psychiatric nurses. In contrast, the program coordinator for mental health emphasised the opposite. According to the participants, more people visit hospitals for mental healthcare than health centres.

*During our supportive supervision, we learned that the outpatient mental health departments were closed during regular clinic hours. Professionals often leave the clinic during clinic hours and complain that mental health services are not in high demand in health centres (IDI #15, program coordinator, government organisation).*

The program coordinators also noted that governments and non-governmental organisations underfund mental health issues and prioritise funding for communicable diseases over mental health issues.

*There are non-governmental organisations that do not fund mental health programs. I am aware of a limited number of leaders of non-governmental organisations who support the mental health initiative. They fund several projects. Nonetheless, very few NGOs provide mental health services when mental health is related to other issues, such as nutrition, HIV, and gender-based violence (IDI #15, a program coordinator in a government organisation).*

## Discussion

Study results suggest that factors influencing mental healthcare and psychosocial services for women experiencing homelessness are incongruous, including divergent intentions, actions, and mismatched problem–solution-resource relationships. Furthermore, there is a lack of gendered and trauma-informed care. Despite that, it is generally acknowledged that women experiencing homelessness face unique challenges.

Participants perceived structural, administrative, and human resource issues as contributing to the service delivery gap for women experiencing homelessness. The Addis Ababa City Administration Bureau of Social and Labour Affairs participants emphasised that the various organisations liaise poorly.

Moreover, the services are overburdened, and the bureau could not address the ever-increasing homelessness and mental health conditions of women experiencing homelessness.

On the other hand, in the capital, mh-GAP is well implemented in primary care settings. However, while patient numbers are high in higher-level public health facilities, low patient flows in primary healthcare centres indicate a mismatch between resource allocation and need. Apart from insufficient coordination among services, the provision of mental health services and psychosocial support are also hindered by prejudice towards people experiencing homelessness.

In our results, prejudice against people experiencing homelessness is visible in contradictory statements of providers expressing compassion and empathy for the care seekers while at the same time considering them to be a security risk and also blaming the clients for their situation. This contradiction is consistent with Heider’s [50] and Weiner’s [51] attribution theory. According to this theory, people interpret their environment in a way that allows them to maintain a positive view of themselves [50]. Failures are attributed to external factors in order to preserve and even enhance a sense of self-worth [52].

In this case, service providers view themselves as without blame, externalising the shortcomings of their service provision to administrative structures and the client’s help-seeking behaviour.

Participants in this study reported that people living in homelessness and/or with mental health conditions are stigmatised and discriminated against. These findings suggest that stigma and discrimination are pervasive in society and are experienced by people living on the streets and/or with mental health conditions. This finding is in accordance with previous research [53].

Stigma and discrimination can lead to further exclusion, marginalisation, and reduced help-seeking and have an impact on employment and estrangement [54, 55]. Research has shown that communities misperceive mental health patients as dangerous [56]. Moreover, they believe that supernatural forces cause mental illnesses and that people with mental health problems have a low recovery rate [56, 57]. In a recent study on the stigma associated with mental illness, individuals with mental health conditions are frequently stigmatised [58]. A study from Ethiopia and a systematic review from the Pacific Rim region corroborated these findings [59, 60].

According to a review article on the consequences of mental health stigma [58], stigma adversely affects self-esteem and self-efficacy, employment and housing, interpersonal relationships, health, treatment adherence and coping, and treatment/seeking behaviour.

Social stigma arises when power is unequal in the social, economic, and political spheres. Thus, people experiencing homelessness are subject to stigmatisation, stereotypes, segregation (us versus them), and discrimination [61]. People may, therefore, not wish to be identified as homeless and/or mentally ill, and fear of discrimination might become an individual or community barrier to seeking help. Homeless clients with mental health conditions are thus less likely to seek care.

The stigma associated with homelessness often promotes violence, criminalisation, and aggressive policies that violate rather than protect human rights [62]. Without a doubt, these marginalised people’s civil, cultural, economic, political, and social rights were violated, including degrading treatment and abuse [62].

In addition, contradictory ideas of protecting and violating human rights were also raised. According to the United Nations, homelessness is an affront to human dignity [63], with Article 22 of the Universal Declaration of Human Rights stating that “every member of society has the right to social security, and is entitled to its realisation through national efforts and international cooperation” [63]. However, participants testified that people experiencing homelessness faced discrimination and human rights violations.

Mental ill health and homelessness are interlinked and require a holistic approach [64]. The study participants noted that most women experiencing homelessness had experience abuse, reproductive health issues, and traumatic experience during their homelessness. However, they failed to consider that women experiencing homelessness lack access to gender-sensitive healthcare and trauma-informed mental health services to help them cope with violence and victimisation. Maximising scarce resources, increasing community awareness of mental health conditions, and humanely approaching those with mental illness and/or experiencing homelessness are also imperative.

Although the study participants emphasised the alarming rate of mental illness, associated burdens, and the lack of attention that is given to people experiencing both homelessness and mental health conditions, some participants pointed to the ongoing provision of food, shelter, and clothing instead of a comprehensive programme that includes mental health services. The tendency to prioritise housing and other basic needs over comprehensive care can be explained by the participants’ belief that meeting essential human needs is paramount.

Health and rehabilitation services and safety were secondary priorities. Very few participants expressed the need to address reproductive health needs. Although the participants pointed out that the problem is complex and that effective treatments are available for mental health conditions and addictions, they rationalised inaction by observing that people without shelter, food, or clothing and those without access to water are less likely to seek psychosocial and mental health treatment. Furthermore, according to the participants, political support for mental health and psychosocial services for women experiencing homelessness is generally short-lived, indicating the difficulty of developing systematic and structured services for such individuals.

This finding aligns with a study conducted in Edinburgh, Scotland, showing how a lack of cognitive and institutional frameworks within homelessness agencies prevents them from providing effective services to individuals with complex needs [25].

A review of the evidence regarding how individuals experiencing homelessness can benefit from health services shows that various models and services are available worldwide. Assertive outreach programmes can help people with mental illness, and supportive programmes can help people with substance addiction.

Recent research has revealed the effectiveness of trauma-informed care (TIC) among individuals experiencing homelessness [65]. Women experiencing homelessness experience trauma, like violence, loss, and relationship break-ups. Besides being homeless, women may feel unstable, unsafe, and disconnected from their community, which can be traumatic.

The emerging model of care in service for homeless victims, TIC [66], rests on the premise that homelessness and associated experiences are traumatic. Like the harm reduction principles of the housing first programme, TIC involves trauma awareness and understanding, an emphasis on safety to avoid re-traumatisation, opportunities to regain control, and a strengths-based approach to focus on clients’ strengths and skill building [66]. As this demonstrates, TIC is an overarching structure and treatment attitude that emphasises understanding, compassion, and response to the effects of all types of trauma [65, 66].

Mental healthcare and psychosocial services for women experiencing homelessness in Addis.

Ababa has been identified as a neglected area of healthcare. Despite addressing women’s specific challenges and the violence and victimisation associated with them, no gender-sensitive or TIC recommendations have been made or are currently offered.

These challenges must be addressed to reduce mental health problems and the adverse effects on mental health of violence. However, participants reported positive aspects of ongoing services, such as non-governmental or governmental organisations’ involvement and memoranda of understanding between charities and hospitals. Although these organisations have different priorities, limited inter-sectionalism and stakeholder availability contribute to closing the service gap.

The study solely relied on the viewpoints and insights of practitioners, and the perspectives of women experiencing homelessness were not sampled (although this work is planned). Moreover, the analysis was conducted without any established theory or framework guiding the process. We performed an inductive thematic analysis. Although inductive thematic analysis is flexible, this flexibility can result in discrepancies and a lack of coherence when developing themes. We systematically worked through all data collection and analysis steps to ensure trustworthiness. We provided in-depth background data to establish the study context. We used purposive sampling in this study in order to cover as many alternative perspectives as possible, and we monitored for data saturation during the data collection process. The raw data was stored in well-organised archives, and all field notes, transcripts, and audio recordings were kept secure. During the interview, sometimes, participants declined to answer specific questions and suggested we contact their managers. During the interview, some participants repeatedly questioned other organisations’ decisions and practices regarding support for people experiencing homelessness. In the study, few participants reflected on their success in providing services and the failures of other leaders and organisations. This behaviour might result from organisational culture and structural factors. Bias may, however, be introduced based on the researchers’ backgrounds and previous professional experience.

## Conclusions

This study suggests that providing mental healthcare and psychosocial services to women experiencing homelessness is hampered by the emergence of controversial and contradictory beliefs and practices on a personal and systemic level. There is a growing need to implement structures and systems to help providers and programme coordinators provide comprehensive, integrated, gender-sensitive, and trauma-informed services to women of reproductive age who are experiencing homelessness, particularly victims of violence, mental health conditions, and reproductive health issues. Therefore, it is crucial to ensure that support services are made available by maximising governance structures, healthcare provision systems, and scarce resources, increasing community awareness of mental health conditions and taking a humane approach towards people experiencing homelessness and mental illness. Furthermore, it must be considered that this study dealt only with providers’ and programme coordinators’ perceptions and experiences over a short period. Therefore, further research on the healthcare needs of women experiencing homelessness and their experience of using health and psychosocial services would be beneficial.

## Electronic supplementary material

Below is the link to the electronic supplementary material.

Supplementary Material 1

Supplementary Material 2

Supplementary Material 3

Supplementary Material 4

## Data Availability

The data underlying this article cannot be made publicly available out of respect for the privacy of the individuals who participated in the study and their roles within the organisations they represent. It is possible to obtain the datasets used and analysed during the current study from the corresponding author upon reasonable request.

## Abbreviations

ICCMH: Integrated Clinical and Community Mental Health
mh-GAP: Mental Health Gap
MHSUA: Mental Health Service Users? Association
PRIME: Programme for Improving Mental Health Care
PTSD: Post-traumatic Stress Disorder
TIC: Trauma Informed Care
