# Supplementary material for: Contradictions hindering the provision of mental healthcare and psychosocial services to women experiencing homelessness in Addis Ababa, Ethiopia: service providers’ and programme coordinators’ experiences and perspectives
Source: BMC Health Serv Res. 2023 Aug 1;23:821. doi: 10.1186/s12913-023-09810-z (PMC10391936; doi:10.1186/s12913-023-09810-z)
Supplement: Supplementary file 2 — Supplementary Material 2 [file 12913_2023_9810_MOESM2_ESM.docx]

| **Process** | **No.** | **Criteria** | **Reported**  **in paper** |
| --- | --- | --- | --- |
| Transcription | 1 | The data have been transcribed to an appropriate level of detail, and the transcripts have been checked against the tapes for ‘accuracy’. | Yes |
| Coding | 2 | Each data item has been given equal attention in the coding process. | Yes |
|  | 3 | Themes have not been generated from a few vivid examples (an anecdotal approach),  but instead the coding process has been thorough, inclusive and comprehensive. | Yes |
|  | 4 | All relevant extracts for all each theme have been collated. | Yes |
|  | 5 | Themes have been checked against each other and back to the original data set. | Yes |
|  | 6 | Themes are internally coherent, consistent, and distinctive. | Yes |
| Analysis | 7 | Data have been analysed / interpreted, made sense of / rather than just paraphrased or described. | Yes |
|  | 8 | Analysis and data match each other / the extracts illustrate the analytic claims. | Yes |
|  | 9 | Analysis tells a convincing and well-organized story about the data and topic. | Yes |
|  | 10 | A good balance between analytic narrative and illustrative extracts is provided. | Yes |
| Overall | 11 | Enough time has been allocated to complete all phases of the analysis adequately, without rushing a phase or giving it a once over lightly. | Yes |
| Written report | 12 | The assumptions about, and specific approach to, thematic analysis are clearly explicated. | Yes |
|  | 13 | There is a good fit between what you claim you do, and what you show you have done / i.e., described method and reported analysis are consistent. | Yes |
|  | 14 | The language and concepts used in the report are consistent with the epistemological position of the analysis. | Yes |
|  |  | The researcher is positioned as active in the research process; themes do not just ‘emerge’. | Yes |

**A 15-point checklist of criteria for good thematic analysis**

Adapted from: Braun, V., & Clarke, V. Using thematic analysis in psychology. Qualitative research in

psychology.2006; 3(2), 77-101.
