# Supplementary material for: Contradictions hindering the provision of mental healthcare and psychosocial services to women experiencing homelessness in Addis Ababa, Ethiopia: service providers’ and programme coordinators’ experiences and perspectives
Source: BMC Health Serv Res. 2023 Aug 1;23:821. doi: 10.1186/s12913-023-09810-z (PMC10391936; doi:10.1186/s12913-023-09810-z)
Supplement: Supplementary file 4 — Supplementary Material 4 [file 12913_2023_9810_MOESM4_ESM.docx]

**Additional file 2: - Interview Guide for healthcare providers and Stakeholders**

In this study, we aim to explore the experiences of healthcare providers and stakeholders in Addis Ababa, Ethiopia, regarding health and psychosocial interventions for street homeless women. This interview aims to get your perspective on how you receive information about the health and psychosocial service delivery situation and your experience with health care and psychosocial support to homeless women. Please remember that there are no right or wrong answers. Feel free to share your thoughts. If there are any issues you do not wish to discuss, you are free not to do so. The interview should last approximately 45 minutes to one hour. All information discussed in the interview is considered confidential. It would be my pleasure to record the interviews with your permission, write down every word from the recordings, and then try to determine what you and the other participants have said. The results of these surveys will be presented in reports. Neither names nor other identifying information will be included in the reports.

**I am grateful for your assistance with this study!**

Participant No -------------------------

Date of Interview ------------------------

Interview Start time ------------------------.

Interview end time -------------------

**Sociodemographic characteristics**

**Annexe II: Topic guides questions**

**Section 1: Background information**

Assigned code for Interviewee: _________

Sex: ___________

Age: ___________

Marital status: ____________

Educational status: ___________

Profession: _______________

Organization: ______________

Department: ________________

Position: ___________________

Location: ___________________

**Interview guide**

1. How do you define homeless people?

A. Please tell me about your views around the concept of homelessness, homeless people from your perspective

------------------------------------------------------------------------------------------------------------------------------------------------------------------------------------------------------------------------------------Notes:- -------------------------------------------------------------------------------

Prompt:

2. What do you know about the current general situation of homeless people in Addis Ababa?

---------------------------------------------------------------------------------------------------------------------------------------------------------------------------------------------------------Notes: - ----------------------------------------------------------------------------------------

**Prompts:**

A. What have you experienced regarding burden over periods in Addis Ababa?

B. Do you have any ideas about patterns of mental health and other disease burden among the homeless?

3. I would like to ask you to describe specific challenges/insecurities, and vulnerabilities for homeless people, specifically homeless women; what do you think about it?

---------------------------------------------------------------------------------------------------------------------------------------------------------------------------------------------------------Notes: - ----------------------------------------------------

Prompts:

A. In general, do you think primary homeless women encounter discrimination?

B. Can you tell me about any difficulties the homeless women may face regarding violence and unmet need? Any other psychosocial and health difficulties?

4. Can you tell me about any policies, guidelines, and strategies in place to support homeless people? Can you explain it, please?

---------------------------------------------------------------------------------------------------------------------------------------------------------------------------------------------------------Notes: - --------------------------------------------------------------------------------------

Prompts: -

A) What kinds of women-specific strategies and policies are currently in place?

B) What would be the best ways to address primary homeless women for you?

5. What are the specific needs of homeless people, particularly homeless women, in Addis Ababa?

A. Can you explain the specific needs regarding financial support, residence place, health care and other needs?

---------------------------------------------------------------------------------------------------------------------------------------------------------------------------------------------------------Notes: - ----------------------------------------------------

6. What guidelines about the fee waiver programme are available that focus on homeless people being eligible for the programme, even without a Kebele ID?

---------------------------------------------------------------------------------------------------------------------------------------------------------------------------------------------------------Notes: - ----------------------------------------------------

7. Are you aware of including service provision to homeless people in the revised Health Extension Manual? How do you explain its relevance?

---------------------------------------------------------------------------------------------------------------------------------------------------------------------------------------------------------Notes: - ----------------------------------------------------

8. What specific services do they provide?

A. Are there actors explicitly targeting the needs of homeless women in Addis Ababa? Can you list and describe each, please?

B. Is there any collaboration between actors? Can you explain it, please?

C. What guides their work?

D. How are they funded?

---------------------------------------------------------------------------------------------------------------------------------------------------------------------------------------------------------Notes: - ----------------------------------------------------

**Part 2- Support for homeless people and mental health service delivery**

How do you describe mental health?

---------------------------------------------------------------------------------------------------------------------------------------------------------------------------------------------------------Notes: - ----------------------------------------------------

How do you explain mental health services and psychosocial support?

---------------------------------------------------------------------------------------------------------------------------------------------------------------------------------------------------------Notes: - ----------------------------------------------------

Are you aware of any policies, guidelines, and strategies in place to support the mental health service neediest?

---------------------------------------------------------------------------------------------------------------------------------------------------------------------------------------------------------Notes: - ----------------------------------------------------

What general services are provided to homeless people in Addis Ababa?

---------------------------------------------------------------------------------------------------------------------------------------------------------------------------------------------------------Notes: - ----------------------------------------------------

What mental health services are provided to homeless women in Addis Ababa?

---------------------------------------------------------------------------------------------------------------------------------------------------------------------------------------------------------Notes: - ----------------------------------------------------

What do you say about the sustainability of these services?

---------------------------------------------------------------------------------------------------------------------------------------------------------------------------------------------------------Notes: - ----------------------------------------------------

Who are the main actors that deliver mental health services for homeless people in Addis Ababa?

---------------------------------------------------------------------------------------------------------------------------------------------------------------------------------------------------------Notes: - ----------------------------------------------------

What specific health and psychosocial services do they provide?

Who are the actors explicitly targeting the needs of homeless women in Addis Ababa? Can you list and describe each, please?

A. How do you explain the collaboration between actors? Can you explain it, please?

B. What guides their work?

C. How are they funded?

----------------------------------------------------------------------------------------------------Notes:

Summary of the Whole interview
